# Effects of *Mollugo pentaphylla* extract on monosodium urate crystal-induced gouty arthritis in mice

**DOI:** 10.1186/s12906-017-1955-1

**Published:** 2017-09-06

**Authors:** Yun Mi Lee, Eun-Jung Shon, Ohn Soon Kim, Dong-Seon Kim

**Affiliations:** Korean Medicine Convergence Research Division, Korea Institute of Oriental Medicine (KIOM), 1672 Yuseong-daero, Yuseong-gu, Daejeon, 34054 South Korea

**Keywords:** Gouty arthritis, Monosodium urate crystal, *Mollugo pentaphylla*, Cytokines, Inflammasome, NF-κB

## Abstract

**Background:**

Gout is an inflammatory condition induced by the deposition of monosodium urate (MSU) crystals in joints and soft tissues, and it can lead to acute or chronic arthritis. MSU are pro-inflammatory stimuli that can initiate, amplify and sustain an intense inflammatory response. In this study, we evaluated the anti-inflammatory effect of an extract of *Mollugo pentaphylla* (MPE) on MSU-induced gouty arthritis in a mouse model.

**Method:**

An MSU crystal suspension (4 mg/50 μL) was injected intradermally into the right paw. The mice were orally administered MPE (150 mg/kg or 300 mg/kg) or the positive control drug colchicine (1 mg/kg) 1 h before the MSU crystals were injected and then once daily for 3 days. The effects of MPE included inflammatory paw edema and pain upon weight-bearing activity, and we evaluated the inflammatory cytokine expression and paw tissue inflammation-related gene expression.

**Results:**

MPE suppressed inflammatory paw edema and pain in the MSU-induced mice. MPE showed anti-inflammatory activity by inhibiting the production of TNF-α, interleukin (IL)-1β, NLRP3 inflammasome and NF-κB.

**Conclusion:**

These results suggest that MPE has potent anti-inflammatory activities and may be useful as a therapeutic agent against gouty arthritis.

## Background

Gout is characterized by increased blood uric acid levels (hyperuricemia) and the deposition of monosodium urate (MSU) crystals within intra-and/or peri-articular areas, which leads to excruciating pain and inflammatory processes [1]. Under long-standing hyperuricemia, MSU crystal deposits further induce chronic inflammatory responses that may lead to joint structure damage, so-called gouty arthritis or chronic gout, which is usually associated with the presence of subcutaneous MSU crystal deposits or tophi [2]. MSU crystals can cause acute, self-limited, inflammatory flares, which are likely triggered by crystal shedding from the cartilage surface into the joint space, where they can interact with resident cells. During attacks of gouty arthritis, MSU crystals induce mass leukocyte infiltration into the joint cavity and are phagocytosed by monocytes/macrophages, resulting in membranolysis and inflammasome activation, which releases interleukin (IL)-1β [3].

The main goals of gouty arthritis therapy are to control pain, reduce inflammatory responses to MSU crystals, and relieve symptoms quickly and safely [4]. Commonly prescribed arthritis medications include nonsteroidal anti-inflammatory drugs (NSAIDs), analgesic drugs, corticosteroids and colchicine [5, 6]. Colchicine is frequently used in the treatment of gout attacks and has specific clinical efficacy and inhibits neutrophil recruitment and activation [7, 8]. Pre-treatment with intravenous colchicine before intra-articular MSU crystal injections greatly reduces inflammation, suggesting that colchicine targets the initial phase of inflammation [9, 10]. However, these agents may have serious side effects, such as gastrointestinal toxicity, renal toxicity, or gastrointestinal bleeding. Natural alternative anti-inflammatory supplements have been used to mediate the inflammatory process and often produce fewer side effects [11]. Therefore, we focused our research on the discovery of a drug with anti-inflammatory activity from natural resources. In our prior research to find novel arthritis drug, we have screened in animal models over 100 plant extracts which are reported on anti-inflammatory effects but not on gouty arthritis.

*Mollugo pentaphylla* (MP) is an annual herb found in the tropical regions of India, Malaysia, China, Japan and Fiji. The aerial parts of this herb are generally consumed for their central nervous system stimulatory as well as their stomachic, aperients, antiseptic, antiperiodic properties, anticancer, antitoxic diuretic, hepatitis and contusion effects. This plant reportedly contains carotenes, vitamin C, and the triterpenoid saponin mollugogenol-A, which exhibits significant antifungal activity [12–15]. In previous reports, Kim et al. showed that MP reduced the *Propionibacterium acnes*-induced secretion of proinflammatory cytokine IL-8 in THP-1 cells, which indicated that MP has anti-inflammatory effects [14]. Lin et al. reported that MP water extracts possess anti-inflammatory activity and can inhibit carrageenan-induced paw edema [16]. Sahu et al. showed that ethanolic extracts of MP significantly reduced the paw edema induced by carrageenan and cotton-pellet induced granuloma models, which are associated with the anti-inflammatory effects in acute and sub-chronic inflammation models. In addition, MP extracts has analgesic activity by tail immersion and acetic acid induced writhing models [17]. Although MP have been showed anti-anflammatory effects and edema suppression effects in animal models, the effects of extract of *Mollugo pentaphylla* (MPE) on MSU crystal-induced gouty arthritis has not been reported. The MSU crystal-induced animal model is widely used to study measurement inflammatory edema related to soft-tissue inflammation and weight-bearing test to estimate pain in gouty arthritis [18]. Considering the protective effect of MP on the anti-inflammatory activity, the study was designed to investigate the anti-inflammatory effect of MPE in MSU crystal-induced gouty arthritis in a mouse model.

## Methods

### MPE preparation

Whole MP plants were collected in Yangpyung, Kyounggi-do. The plant materials were confirmed taxonomically by Dr. Geung-Joo Lee of the Chungnam National University. A voucher specimen (no. KIOM201701018962) was deposited at the Korean herbarium of Standard Herbal Resources at the KIOM. A dried whole MP plant (1 kg) was extracted twice with 70% ethanol (with a 3 h reflux), and the extract was then concentrated under reduced pressure. The decoction was filtered, lyophilized, and stored at 4 °C [19]. The yield of the dried extract from crude starting materials was 13.9% (*w*/w).

### MSU crystal synthesis

4 g of uric acid was dissolved and heated in 800 ml of H_2_O with NaOH (9 ml/0.5 M) and adjusted to pH 8.9 at 60 °C. The solution was cooled overnight in a cold room. The crystals were harvested by decanting the supernatant and then washed and dried. The needle shape and size of the crystals were checked by polarizing light microscopy. The MSU crystals were suspended in 2.5% Tween 80 in PBS (80 mg/ml) [20].

### Induction of gouty arthritis with MSU crystals in mice

C57BL6 male mice (7 weeks old, 20-22 g body weight) were purchased from Orient Bio, Seongnam, Korea), The mice were housed in a controlled temperature room at 22 ± 2 °C, in 55 ± 10% relative humidity with a 12 h: 12 h light-dark cycle, and they were freely fed commercial standard chow (Dae-Han Bio Link) and were provided tap water ad libitum for 6 days. After acclimation, the mice were housed separately in cages and were familiarized with the testing procedures. C57BL6 male mice (8 weeks old, 20-22 g body weight) were divided into the following five groups, which consisted of five animals each: (1) control group, (2) MSU crystal group with MSU injection, (3, 4) the MPE-treated group (150, 300 mg/kg body weight, respectively) with MSU crystal injection and (5) the colchicine (Col) treated group (1 mg/kg body weight) with MSU crystal injection. The MSU crystal suspension (4 mg/50 μL) was injected intradermally into the right paw. The mice were administered MPE or the positive control drug (colchicine) 1 h before the MSU crystal injection and then once daily for 3 days. After the treatment with MPE, no evidence of systemic adverse effects was observed in any study group. Four days after MSU crystal injection, the mice were anesthetized using Pentobarbital sodium (Entobal, Hanlim Pharma, Co., Ltd., Korea) and sacrificed. The right paw tissue was removed and homogenized at 4 °C in RIPA buffer. The homogenate was centrifuged at 14000 rpm for 15 min at 4 °C, and the supernatant was stored at −70 °C until further analysis. All experiments that used animals were approved by the Institutional Animal Care and Use Committee of the Korea Institute of Oriental Medicine (Daejeon, Korea) and was conducted in accordance with the Guide for the Care and Use of Laboratory Animals published by the US National Institutes of Health (Bethesda, MD, United States).

### Assessment of inflammatory paw edema

Inflammatory paw edema was quantified by measuring the thickness of the paw with a Vernier scale at the end of the experimental period.

### Assessment of inflammatory pain

After arthritis induction, the original weight-bearing capability balance of the hind paws was disrupted. A significant shift of weight from the arthritic site to the contralateral limb, i.e., a weight-bearing deficit, is considered a pain measure and has been shown in joint arthritis models induced by intra-articular MSU crystals [21]. The inflammatory pain was quantified by measuring the weight-bearing capacity of a paw load using a dynamic weight bearing (DWB) device (Bioseb, Boulogne, France) [22, 23]. The weight distribution ratio was calculated by the following equation: [weight on right hind limb / (weight on right hind limb + weight on left hind limb)] × 100.

### Measurement of inflammatory cytokines

The levels of IL-1β and TNF-α were measured using ELISA kits from R&D Systems (Minneapolis, MN, USA) according to the manufacturer’s protocol.

### Real-time PCR analysis

Total RNA was isolated using TRIzol (Invitrogen, CA, USA), and 0.5 μg of total RNA was reverse transcribed into cDNA with the PrimeScript First Strand cDNA Synthesis kit (Bio-Rad, CA, USA). Real-time quantitative PCR was performed with specific primers using a CFX Connect™ Real-Time PCR Detection System (Bio-Rad, CA, USA). The primer sequences are shown in Table 1. All real-time PCR experiments were run in quadruple. The mRNA levels of GAPDH were determined for the normalization of the NLRP3, ASC, NF-κB, caspase-1, IL-1β and TNF-α mRNA expression values using the CFX Manager™ Software (Bio-Rad, CA, USA).

**Table 1:** Real-time PCR primer sequences

Gene		Primer sequence
IL-1β	Forward	5′-TCTATACCTGTCCTGTGTAATGAAAG-3′
	Reverse	5′-GGCTTGTGCTCTGCTTGTGAG-3′
TNF-α	Forward	5′-GTGGAACTGGCAGAAGAG-3′
	Reverse	5′-CCATAGAACTGATGAGAGG-3′
NF-κB	Forward	5′-CTCACCGGCCTCATCCACAT-3′
	Reverse	5′-TGGCTAATGGCTTGCTCCAG-3′
NALP3	Forward	5′-TGCTCTTCACTGCTATCAAGCCCT-3′
	Reverse	5′-ACAAGCCTTTGCTCCAGACCCTAT-3′
ASC	Forward	5′-CAGAGTACAGCCAGAACAGGACAC-3′
	Reverse	5′-GTGGTCTCTGCACGAACTGCCTG-3′
Caspase-1	Forward	5′-TCCGCGGTTGAATCCTTTTCAGA-3′
	Reverse	5′-ACCACAATTGCTGTGTGTGCGCA-3′
GAPDH	Forward	5′-AGAAGGTGGTGAAGCAGGCATC-3′
	Reverse	5′-CGAAGGTGGAAGAGTGGGAGTTG-3′

### Statistical analysis

The results were expressed as the mean ± standard error of the mean (SEM) and analyzed using a one-way analysis of variance (ANOVA) followed by Dunnett’s tests for multiple comparisons or unpaired Student’s *t-*tests for two-group comparisons. All analyses were performed using *Prism* 7.0 (GraphPad Software, San Diego, CA, USA), and *P*-values <0.05 were considered statistically significant.

## Results

### Effect of MPE on MSU crystal-induced paw edema

**Fig. 1:**
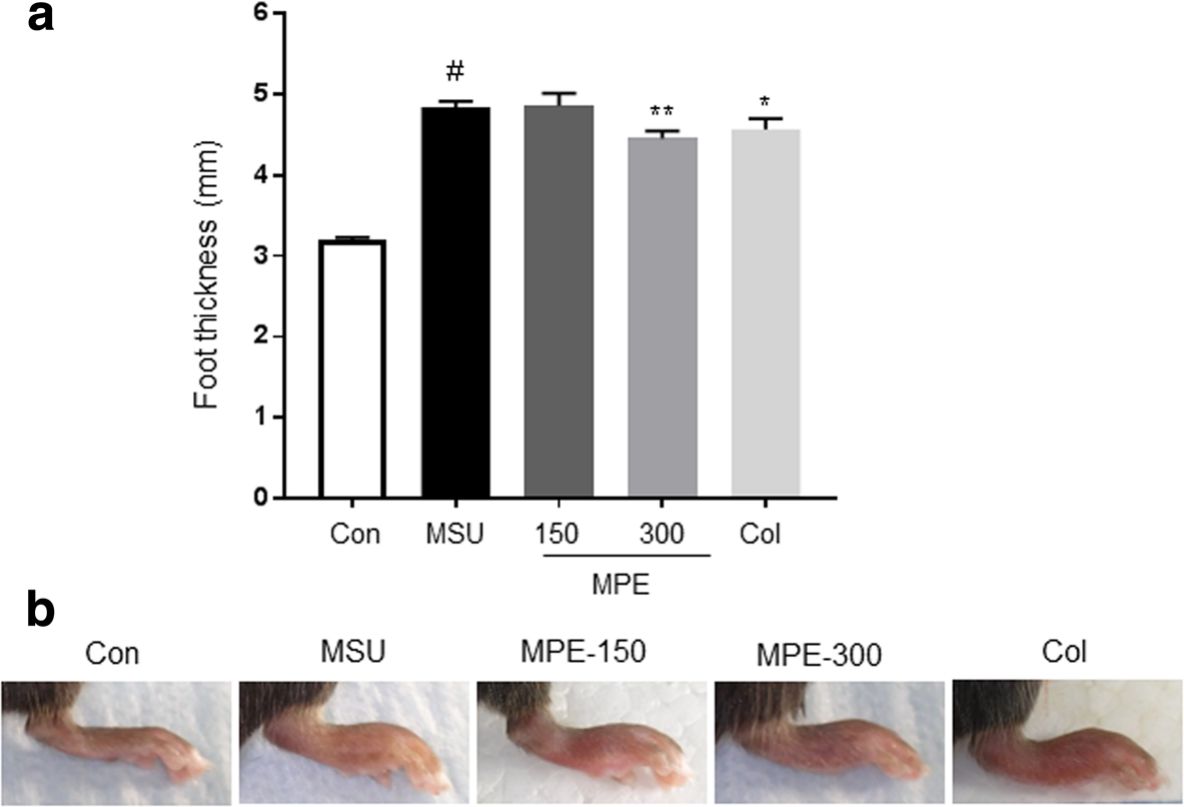
Effect of MPE on paw edema in mouse with MSU crystal-induced gouty arthritis. Con, control mice; MSU, MSU crystal-induced mice; MPE-150, MSU mice treated with 150 mg/kg of MPE; MPE-300, MSU mice treated with 300 mg/kg of MPE; Col, MSU mice treated with 1 mg/kg of colchicine. **a** Measurement of the thickness of the each mice paw recorded at the end of the experimental period. **b** Representative images of the left leg from each group are shown. Data are presented as the mean ± SEM (*n* = 5). # *p* < 0.0001, vs. control group; ** *p* < 0.01, vs. MSU group, * *p* < 0.05, vs. MSU group

To assess the extent of edema, the thickness of the paws of the control and treated mice was measured. As shown in Fig. 1a and b, the MSU crystals in the treatment mice led to a significant increase in the foot thickness compared with that of the control mice; however, the increase in foot thickness was found to be reduced in the MSU crystal-induced mice treated with MPE (300 mg/kg) and colchicine. These results suggest that MSU crystal-induced paw edema was suppressed by MPE.

### Effect of MPE on hind paw weight-bearing distribution

**Fig. 2:**
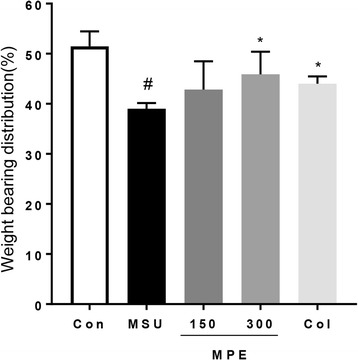
Effect of MPE on changes in hind paw weight-bearing distribution in MSU-induced gouty arthritis. The weight-bearing distribution ratio was measured using a dynamic weight bearing (DWB) device, compared to that of the MSU crystal-induced group. Con, control mice; MSU, MSU crystal-induced mice; MPE, MSU mice treated with 150/300 mg/kg of MPE; Col, MSU mice treated with 1 mg/kg of colchicine. Data are presented as the mean ± SEM (*n* = 5). # *p* < 0.001, vs. control group; * *p* < 0.05, vs. MSU group

The ratio of hind paw weight distribution between the right and left limbs was used to assess the progressive pain of gouty arthritis. The weight-bearing distribution was reduced in the MSU crystal group mice compared with that in the control group mice, although the MPE-treated group and Col-treated groups displayed a significant reversal of this MSU crystal-induced inflammatory pain in DWB measurements (Fig. 2). Consequently, the MPE treatment helped relieve the pain of MSU crystal-induced gouty arthritis.

### Effects of MPE on proinflammatory cytokines

**Fig. 3:**
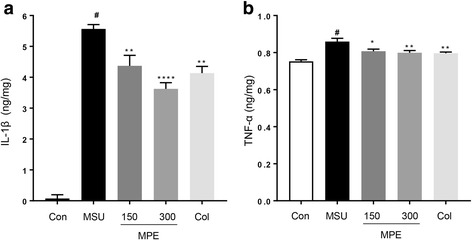
Effects of MPE on expression levels of inflammatory cytokines in MSU crystal-induced gouty arthritis in mouse paw tissue. Con, control mice; MSU, MSU crystal-induced mice; MPE, MSU mice treated with 150/300 mg/kg of MPE; Col, MSU mice treated with 1 mg/kg of colchicine. **a** IL-1β and **b** TNF-α levels were measured by ELISA. Data are presented as the mean ± SEM (*n* = 5). # *p* < 0.0001 vs. control group; * *p* < 0.05, ** *p* < 0.01 and **** *p* < 0.0001 vs. MSU group

We investigated the anti-inflammatory effect of MPE on TNF-α and IL-1β. The levels of these two cytokines were examined via ELISA after the injection of the MSU crystal suspension. The results showed (Fig. 3) that MSU-induced mice had significantly elevated TNF-α and IL-1β levels and that treatment with MPE significantly down-regulated the production of TNF-α and IL-1β. Colchicine also significantly decreased the proinflammatory cytokine levels compared with those in the MSU group. These results clearly showed that the MPE treatment inhibits the major inflammatory cytokines (TNF-α and IL-1β), which are essential in the initiation and progression of gouty arthritis.

### Real-time PCR analysis

**Fig. 4:**
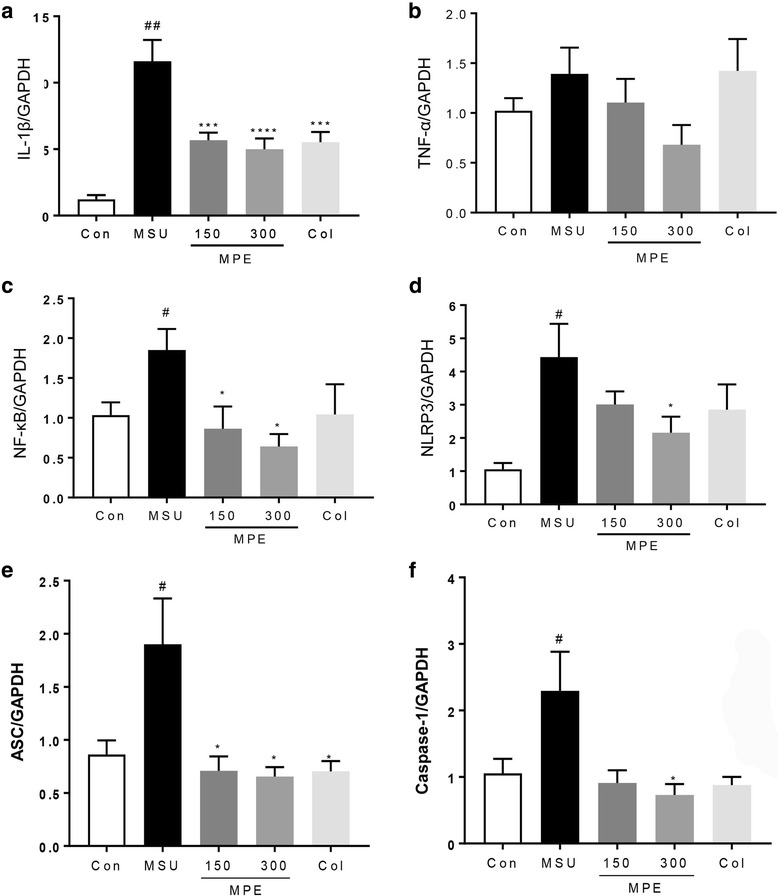
Effects of MPE on the expression of cytokines in the paw tissue of mouse with MSU crystal-induced gouty arthritis. Con, control mice; MSU, MSU crystal-induced mice; MPE, MSU mice treated with 150/300 mg/kg of MPE; Col, MSU mice treated with 1 mg/kg of colchicine. Expression of **a** IL-1β, **b** TNF-α, **c** NF-κB, **d** NALP3, **e** ASC and **f** Caspase-1 mRNA was determined by real-time RT-PCR. The mRNA expression levels were normalized to GAPDH mRNA level. Data are presented as the mean ± SEM (*n* = 5). #*p* < 0.05 and ##*p* < 0.0001 vs. control group; * *p* < 0.05, *** *p* < 0.001 and **** *p* < 0.0001 vs. MSU group

To investigate the mechanisms underlying the anti-inflammatory effects of MPE, the mRNA expression levels of inflammatory cytokines (TNF-α and IL-1β), inflammasome components (NLRP3, ASC, caspase-1) and transcription factors (NF-κB) were determined in MSU-induced paw tissue via RT-PCR. As shown in Fig. 4, a marked increase in the mRNA expression levels of inflammatory cytokines (TNF-α and IL-1β), NLRP3, ASC, caspase-1, and NF-κB was observed in the paw tissue of MSU-induced mice compared with the levels observed in the control group. Conversely, compared with the MSU group, the MSU group treated with MPE showed a dose-dependent decrease in the transcriptional level of inflammatory cytokines (TNF-α and IL-1β), NLRP3, ASC, caspase-1, and NF-κB. These findings indicate that the decreased mRNA levels of NLRP3, ASC, caspase-1 and NF-κB are responsible for the reduction in cytokine production.

## Discussion

Gouty arthritis is a chronic inflammatory disease characterized by swelling and severe pain of one or more synovial joints, and it is caused by disordered nucleic acid metabolism and the subsequent deposition of MSU crystals in the joints [24]. MSU crystals are proinflammatory stimuli that can initiate, amplify and sustain an intense inflammatory response. The main pathological hallmark of gout is that neutrophils accumulate in both the joint fluid and the synovial membrane after sensing the signals released from macrophages. The interaction of MSU crystal with the macrophages in the joints leads to the formation of proteins known as inflammasomes, which provide a platform for the enzymatic conversion of pro-IL-1β into biologically active IL-1β. Then, the activated IL-1β promotes TNF-α, which is responsible for the significant influx of neutrophils into both the joint fluid and the synovial membrane [25, 26]. Activated TNF-α and IL-1β amplify the inflammatory response and cause joint injury, and they are also activators of NF-κB [27, 28]. Thus, the activation of NF-κB may be a key step in the pathogenesis of gouty arthritis, and the suppression of NF-*κ*B likely represents an effective treatment for the treatment of gouty arthritis [29]. Therefore, the important targets for the management of inflammatory diseases, such as gouty arthritis include the reduction of swelling, pain, and inflammation by controlling the activation of inflammasomes, proinflammatory cytokines (TNF-α and IL-1β) and NF-κB [4, 30, 31].

In this study, we examined the anti-inflammatory effect of MPE on the production of proinflammatory cytokines (TNF-a and IL-1β), which are mediators of inflammation-related gene expression in the MSU crystal-induced mice. We further evaluated the therapeutic potential of MPE for the treatment of the anti-inflammatory agent by measuring the paw thickness and weight-bearing distribution in the paw tissue of MSU-induced mice.

A number of studies have demonstrated that proinflammatory cytokines including IL-1β and TNF-α, and the transcription factor, NF-κB, are important in the response to MSU injections into the joint cavity [28, 32]. The proinflammatory cytokines TNF-α and IL-1β both activate NF-κB and are activated by NF-κB [33]. NF-κB is a dimeric transcription factor that appears to play a major role in the regulation of inflammatory gene expression. However, under the influence of extracellular signals, such as MSU, NF-κB can be activated and may increase the expression of genes for proinflammatory cytokines, chemokines, enzymes, and adhesion molecules, which ultimately leads to an inflammatory response closely connected to the pathogenesis of gouty arthritis. Jaramillo et al. reported that MSU-induced NF-κB activation and proinflammatory cytokine secretion in the murine macrophage cell line B10R [34]. Conversely, the overexpression of TNF-α and IL-1β can directly activate the NF-κB pathway [28, 29]. Han et al. showed that NF-κB are simultaneously activated in IL-1 or TNF-α stimulated synoviocytes as well as the intimal synovial lining of Rheumatoid arthritis patients [35]. Our results show that MPE inhibits the production of TNF-α and IL-1β in MSU crystal-induced mice. The levels of IL-1β and TNF-α in the paw tissue were significantly increased in response to MSU, whereas this overproduction was markedly decreased by the MPE treatment, compared to the positive drug, colchicine. Additionally, the findings in the present study demonstrated that MPE prevented the MSU-induced activation of NF-κB.

Activated NF-κB promotes the expression of NLRP3 [36]. Recent studies have reported that epigallocatechin-3-gallate can inhibit NLRP3 inflammasome activation by blocking NF-κB activation [37]. The NLRP3 (also known as NALP3) inflammasome has been shown to form through homotypic interactions between the CARD and PYD domains of NLRP3, pyrin and the adaptor ASC (Apoptosis-associated speck-like protein containing a CARD). After MSU stimulation, the NLRP3 inflammasome is activated and induces the conversion of procaspase-1 to active caspase-1, which in turn cleaves the inactive precursor cytokine pro-IL-1β into proinflammatory IL-1β [38–40]. Kingsbury et al. suggested that macrophages from mice deficient in various components of the inflammasomes including NLRP3, ASC, and caspase-1, could not activate IL-1β in response to MSU stimulation [41]. In the present study, we showed that the gene expression levels of NLRP3, ASC and caspase-1 in MSU-induced mouse paw tissue were reduced after administering MPE. The decreased expressions of NALP3 by MPE further restricted the activation of caspase-1. The production of the mature forms of IL-1β and TNF-α were also decreased. We also provide evidence for a gouty arthritis-related pain-relieving effect of MPE, which significantly reduces edema and weight-bearing pain in MSU-induced mice. Collectively, our results suggest that the inflammatory and gouty arthritis inhibitory effects of MPE primarily occur via the down-regulation of edema, pain and inflammation through the control of inflammasomes, proinflammatory cytokines (TNF-α and IL-1β), and NF-κB.

## Conclusion

The present study suggests for the first time that MPE suppressed MSU crystal-induced swelling and pain in mouse and exerted anti-inflammatory effect via suppressing proinflammatory cytokines (TNF-α and IL-1β), NLRP3 inflammasome and NF-κB activation. These findings indicate the potential therapeutic effect of MPE that may inhibit gouty arthritis through blocking multiple anti-inflammatory pathways and reducing swelling and pain.

## Abbreviations

MSU: Monosodium urate
MP: *Mollugo pentaphylla*
MPE: Extract of *Mollugo pentaphylla*
IL-1β: Interleukin-1 beta
TNF-α: Tumor necrosis factor-alpha
NLRP3: NACHT, LRR and PYD domains-containing protein 3
ASC: Apoptosis-associated speck-like protein containing a CARD
NF-κB: Nuclear factor kappa-light-chain-enhancer of activated B cells
